# Comparison of clinical-CT segmentation techniques for measuring subchondral bone cyst volume in glenohumeral osteoarthritis

**DOI:** 10.1186/s40634-022-00564-x

**Published:** 2023-01-06

**Authors:** Aoife M. R. Pucchio, Nikolas K. Knowles, Joan Miquel, George S. Athwal, Louis M. Ferreira

**Affiliations:** 1 Department of Surgery, Schulich School of Medicine and Dentistry, Western University, London, ON Canada; 2 Department of Biomedical Engineering, Western University, London, ON Canada; 3 Roth|McFarlane Hand and Upper Limb Centre, St. Joseph’s Health Care, London, ON Canada; 4 Department of Kinesiology and Health Sciences, University of Waterloo, Waterloo, ON Canada; 5 Department of Mechanical and Materials Engineering, Western University, 1151 Richmond St, London, ON Canada

**Keywords:** Cystic osteoarthritis, Subchondral bone cysts, Segmentation, Thresholding, Region Growing, Clinical-CT

## Abstract

**Purpose:**

This study aimed to assess the accuracy and reproducibility of four common segmentation techniques measuring subchondral bone cyst volume in clinical-CT scans of glenohumeral OA patients.

**Methods:**

Ten humeral head osteotomies collected from cystic OA patients, having undergone total shoulder arthroplasty, were scanned within a micro-CT scanner, and corresponding preoperative clinical-CT scans were gathered. Cyst volumes were measured manually in micro-CT and served as a reference standard (*n* = 13). Respective cyst volumes were measured on the clinical-CT scans by two independent graders using four segmentation techniques: Qualitative, Edge Detection, Region Growing, and Thresholding. Cyst volume measured in micro-CT was compared to the different clinical-CT techniques using linear regression and Bland–Altman analysis. Reproducibility of each technique was assessed using intraclass correlation coefficient (ICC).

**Results:**

Each technique outputted lower volumes on average than the reference standard (-0.24 to -3.99 mm^3^). All linear regression slopes and intercepts were not significantly different than 1 and 0, respectively (*p* < 0.05). Cyst volumes measured using Qualitative and Edge Detection techniques had the highest overall agreement with reference micro-CT volumes (mean discrepancy: 0.24, 0.92 mm^3^). These techniques showed good to excellent reproducibility between graders.

**Conclusions:**

Qualitative and Edge Detection techniques were found to accurately and reproducibly measure subchondral cyst volume in clinical-CT. These findings provide evidence that clinical-CT may accurately gauge glenohumeral cystic presence, which may be useful for disease monitoring and preoperative planning.

**Level of evidence:**

Retrospective cohort Level 3 study.

**Supplementary Information:**

The online version contains supplementary material available at 10.1186/s40634-022-00564-x.

## Background

Cyst formation is a characteristic finding in end-stage osteoarthritis (OA), along with subchondral sclerosis, joint space narrowing, and abnormal bone formation [2]. Cystic changes in glenohumeral OA compromise the integrity of subchondral bone, leading to clinical complications following joint replacement surgery. In symptomatic end-stage OA, the articular surfaces of the joint are often surgically replaced with metallic or polyethylene components that depend on the underlying bone for stability, fixation, and support. As fixation relies partially on bony ingrowth for stability, structural changes within the glenohumeral joint such as cysts may impact the survival of the implant. Fixation may be further compromised with stemless humeral implants that minimize bone removal, given that cysts may extend to the osteotomized bone surface. Glenohumeral subchondral bone cyst presence has been correlated to increased risk of early revision after total shoulder arthroplasty [14].

With the widespread availability of commercial image analysis software, quantification of bone characteristics has become increasingly common. In-vivo measurement of subchondral bone cysts using preoperative clinical-computed tomography (CT) scans can allow for the quantification of OA severity, progression, and cystic presence. This may be a useful tool to monitor OA progression, inform surgical planning, and preoperatively assist in determining risk of implant failure. Tanner et al. (2018) concluded that evaluating preoperative CT scans for cystic presence may be useful to determine if these cystic changes could affect implant survivorship [14]. Despite CT being routinely used, the validity of subchondral bone cyst volume measurements in clinical-CT has not been established.

The objective of this study was to assess the accuracy and reproducibility of four common commercially available segmentation techniques (Qualitative, Edge Detection, Region Growing, and Thresholding) for measuring subchondral bone cyst volume in clinical-CT scans of glenohumeral OA patients, using co-registered micro-CT images as the reference standard. Given the lower resolution of clinical-CT, we hypothesized that algorithms which rely on small feature identification (i.e. Qualitative and Edge Detection) would have lower accuracy and reliability compared to algorithms based on larger area grayscale contrast (i.e. Regional Growing and Thresholding), and thus will have lower accuracy compared to the micro-CT reference standards.

## Materials and methods

### Sample acquisition and imaging

This was a retrospective analysis of a CT database of images from approximately 30 total shoulder arthroplasty patients. This database included both preoperative clinical-CT scans and postoperative micro-CT scans of the humeral head osteotomies acquired at surgery. These CT scans were assessed for the presence of cysts, and we excluded scans that had cysts with undefined boundaries, including cysts that went into the articular surface and cysts that bled into each other. We found that 10 patients had mild to moderate cystic presence with subchondral bone cysts that were suitable for measurement. Thus, the inclusion criteria was osteoarthritis with measurable cysts in the region of the humeral head osteotomy.

The ten selected humeral head osteotomies, excised at the head to neck junction, were collected from a subset of patients with primary end-stage osteoarthritis who had previously undergone total shoulder arthroplasty (mean age: 65 ± 12 years old; 7 males; 3 females). Each humeral head osteotomy was scanned using a micro-CT scanner (Nikon XT H 225 ST, Nikon Metrology, Cambridge, ON, Can; 20 µm isotropic voxels, 95 kV, 80 µA, 3141 projections, 1000 ms exposure). Corresponding preoperative clinical-CT scans (GE Discovery CT750 HD, Milwaukee, WI, USA; pixel dimension 0.527–0.645 mm, slice thickness 1.25 mm, 120 kVp, 200 mA) for each patient were gathered retrospectively from patient charts. The micro-CT osteotomy was registered to the respective clinical-CT scan by iterative closest point registration of the bone [9]. This study was approved by the Health Sciences Research Ethics Board at Western University (HSREB# 113,023).

### Image analysis

**Fig. 1:**
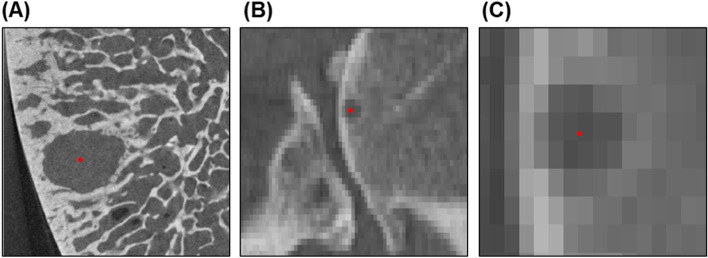
Process of cyst identification in humoral head osteotomies. **A** Cysts were identified on micro-CT scan, as denoted by the red dot. **B** Corresponding cyst identified on preoperative clinical-CT scan. **C** Zoomed in view of cyst in clinical-CT. Note the cyst boundaries are not clearly defined

Cysts were defined as an elliptical, spherical or irregularly shaped volume > 1.25 mm in diameter of lower grayscale (lower Hounsfield unit [HU]) surrounded by an area of higher grayscale (higher HU) [4, 6, 12]. Individual cysts were identified on the micro-CT scan for measurement in this study (Fig. 1). If multiple cysts were identified in a subject, each were treated and analyzed independently as separate cases for comparison between segmentation methods; two subjects had multiple cysts, providing a total of 13 cysts to be evaluated (*n* = 13). Identified cysts were manually measured in micro-CT using free hand segmentation aided by automated edge detection, similar to a previously introduced gold standard segmentation technique [10] (Mimics v.20.0, Materialise, Leuven, BE). These micro-CT volume measurements served as the reference standard to which the clinical-CT segmentation techniques were compared.

**Fig. 2:**
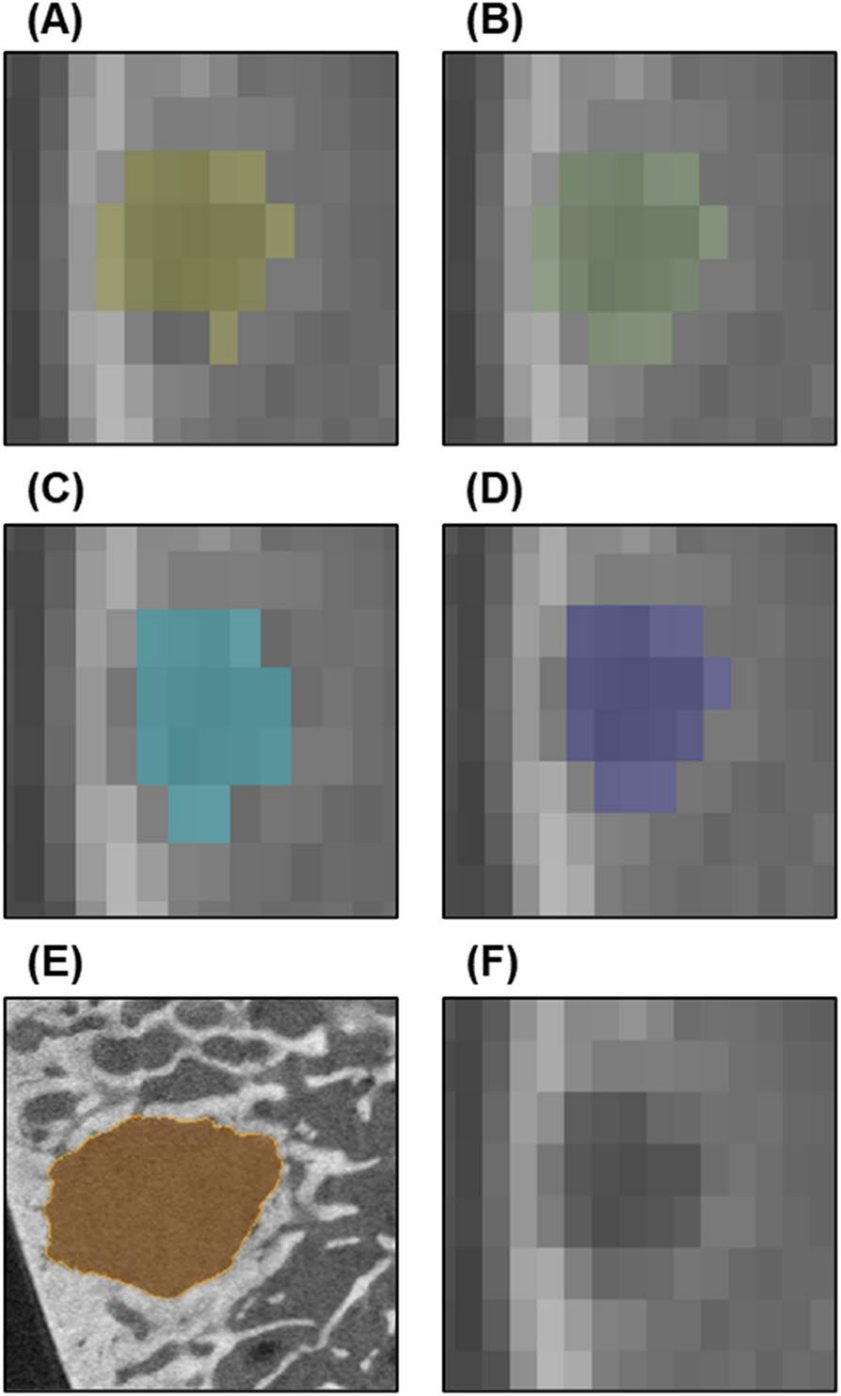
Visual comparison of the different clinical-CT segmentation techniques. **A** Edge Detection. **B** Qualitative. **C** Region Growing. **D** Thresholding. **E** Cyst as measured on micro-CT, that served as the reference standard for volume measurement comparisons. **F** Cyst that was measured by the four clinical-CT segmentation techniques as pictured on clinical-CT

Corresponding cyst volumes were measured in clinical-CT using four different techniques: Qualitative, Edge Detection, Region Growing, and Thresholding (Fig. 2). Cysts were segmented using the built-in image processing tools in commercially available software (Mimics v.20.0). These techniques were selected based on their prevalence in image analysis software and reported use in previously published studies [5, 14, 15]. All clinical-CT segmentation measurements were completed by two independent, blinded graders (JM & AP). A fellowship-trained shoulder surgeon completed the measurements (JM), as well as a researcher (AP) trained by senior researchers with guidance from a radiographic atlas [1] and previous literature evaluating cysts using CT scans. Volume data were collected following all measurements to reduce bias.

Qualitative measurements consisted of slice-by-slice freehand segmentation of voxels qualitatively identified to be part of the cyst by the grader based on their relative intensity. Edge Detection worked by semi-automated determination of the cyst boundary using object contours automatically computed using gradient magnitude and manually placed points on multiple planes of the scan. Region Growing worked by extracting all connected voxels from a manually placed seed point guided by cyst-specific thresholds defining the midpoint HU value of the boundary between cyst interior and surrounding bone, similar to the half maximum height threshold technique [13]. Thresholding is a technique where every voxel below a specified intensity value is selected. The mean cyst-specific midpoint intensity value calculated for Region Growing measurements was used as a universal threshold value for all Thresholding measurements. Segmentation was completed primarily on the axial and coronal planes. Detailed descriptions of the segmentation methods used are provided as an additional supporting file (Additional file 1: Table S1).

### Statistical analysis

Measurement techniques were assessed on two parameters: accuracy to micro-CT standard and reproducibility, with cyst volumes measured in micro-CT used as the standard for comparison. Statistical analysis was performed in SPSS 26.0 (IBM Corp., Chicago, IL). The CT database from which this retrospective analysis was derived was a representative sample of osteoarthritic shoulder arthroplasty patients at our surgical centre, and the final number of 10 patients included in this study represents the incidence of mild to moderate cystic OA within the original group.

Descriptive statistics (mean, standard deviation [SD], and range) of cyst volumes measured in clinical-CT are reported. Accuracy of each technique relative to the micro-CT standard volume measurements was assessed using linear regression and Bland–Altman analysis, as volume measurements are continuous variables. Values of *p* < 0.05 were considered statistically significant. Root mean square error (RMSE) from the micro-CT standard was also calculated to assess accuracy. The reproducibility of each technique was assessed by calculating intraclass correlation coefficient (ICC) for the inter-observer measurements based on a single rating, consistency, 2-way mixed-effects model. The 95% CI of the ICC estimates were used, with values > 0.90 indicating excellent reliability, values between 0.75 and 0.9 indicating good reliability, and values between 0.5 and 0.75 indicating moderate reliability [8].

## Results

Thirteen subchondral bone cysts in cystic OA subjects were evaluated. Individual cyst volumes ranged from 5.60 mm^3^ to 69.45 mm^3^, with a mean of 21.54 mm^3^ (SD = 17.41). Cyst-specific midpoint HU values used to guide Region Growing measurements had a range of 164 to 531, with a mean of 392.

**Fig. 3:**
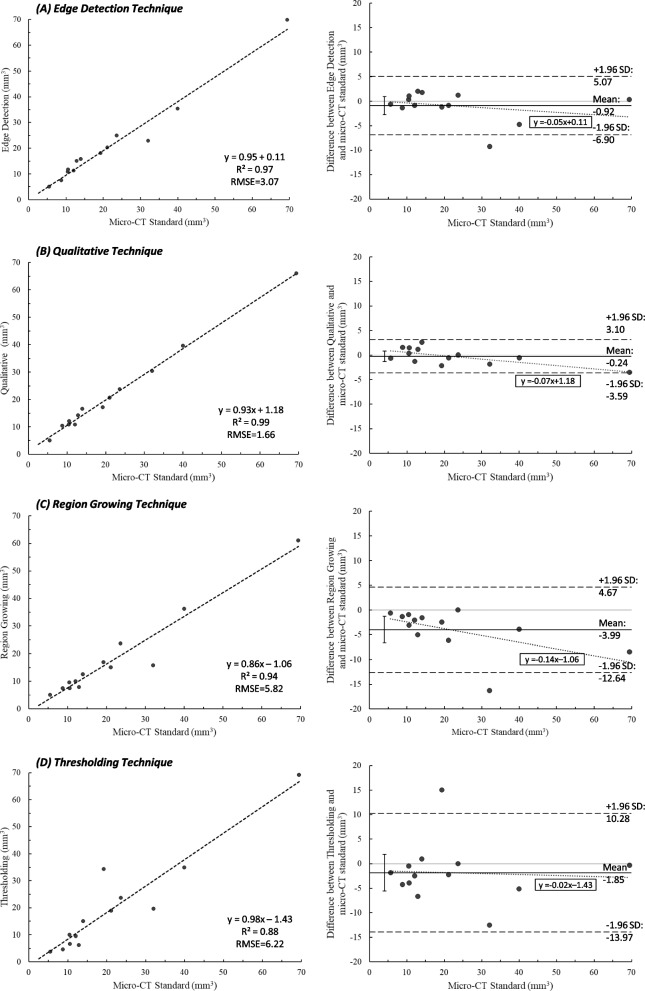
Linear regression and Bland–Altman of cyst volume (mm^3^) measured using four clinical-CT segmentation techniques (**A**-**D**). All linear regression slopes and intercepts were not significantly different than 1 and 0, respectively (*p* < 0.05). In Bland–Altman plots, solid line indicates mean bias, dashed lines indicate upper and lower limits of agreement, and solid gray line indicates line of no bias. Linear regression of Bland–Altman plot shown in gray small-dotted line

Cyst volume measured manually in micro-CT was used as a standard to compare the four different clinical-CT segmentation techniques using linear regression and Bland–Altman analysis (Fig. 3; Table 1). All methods on average produced lower volumes than the standard. The smallest bias was observed for Qualitative segmentation, with a mean difference of 0.24 ± 1.71 mm^3^ from the standard. Region Growing resulted in the largest bias, with a mean difference of 3.99 ± 4.42 mm^3^ from the standard. The 95% confidence interval (CI) for slope and intercept of the linear regression encompassed one and zero, respectively, for all methods (*p* < 0.05). In the Bland–Altman analysis, the 95% CI of mean difference for Qualitative, Edge Detection and Thresholding measurements encompassed zero, and the Qualitative and Edge Detection measurements also showed a relatively even spread around zero. Qualitative and Edge Detection segmentation resulted in relatively narrow limits of agreement, while Region Growing and Thresholding segmentation displayed wider limits of agreement. Overall accuracy to the micro-CT standards was met best by Qualitative and Edge Detection, respectively.

**Table 1:** Accuracy (with standard deviation [SD]) of clinical-CT segmentation techniques compared to micro-CT reference standards

Segmentation Technique	Mean difference ± SD (mm^3^)	Root mean square error (mm^3^)
Edge Detection	-0.92 ± 3.05	3.073
Qualitative	-0.24 ± 1.71	1.657
Region Growing	-3.99 ± 4.42	5.822
Thresholding	-1.85 ± 6.18	6.222

The 95% CI of the ICC estimate calculated for inter-observer measurements (Table 2) was good to excellent for Qualitative and Edge Detection, moderate to excellent for Thresholding, and poor to excellent Region Growing. Agreement between observers followed similar patterns to that of accuracy, with methods yielding the smallest mean differences demonstrating the highest inter-observer reliability, and reliability decreasing as mean difference increased.

**Table 2:** Intraclass correlation coefficient (ICC) for inter-observer agreement (95% confidence interval [CI])

Segmentation Technique	ICC (95% CI)
Edge Detection	0.92 (0.76, 0.98)
Qualitative	0.93 (0.79, 0.98)
Region Growing	0.81 (0.48, 0.94)
Thresholding	0.88 (0.65, 0.96)

## Discussion

Cystic changes in glenohumeral OA may compromise the integrity of subchondral bone and impact survivability of arthroplasty components. While CT imaging has previously been used to measure cyst volumes [5, 14, 15], clinical-CT techniques for quantifying cyst volume in glenohumeral OA have not been validated. This study demonstrated that Qualitative and Edge Detection segmentation techniques may accurately reflect cyst volumes in the glenohumeral joint, which refutes the hypothesis that techniques involving small feature identification would perform poorly with clinical resolution CT scans. These segmentation techniques also showed adequate precision demonstrated by their narrow limits of agreement, and were found to have good to excellent reproducibility between graders. The 95% CI of mean difference encompassing zero for these techniques, and the relatively even spread of measurements around zero, indicate an absence of systematic bias from the micro-CT standard for these techniques.

Tanner et al. (2018) quantified subchondral bone cyst volume in the glenohumeral joint using clinical-CT by segmenting cysts based on grayscale values below the non-cortical bone equivalent [14], similar to the Thresholding technique used in this paper. While they reported good agreement between these measurements and qualitative categorizations of the level of cyst formation (none, small, medium, large), findings in our study may suggest that subchondral cysts can contain grayscale values higher than the non-cortical bone equivalent, as the mean HU value defining the boundary between cyst interior and surrounding bone was found to be 392 (compared to a value of approximately 300–400 for non-cortical bone). This is likely due to partial volume averaging caused by differences in clinical-CT imaging parameters between studies. The use of a calibration phantom to accurately quantify bone mineral density (BMD) may provide a more accurate threshold across studies, but is seldom used in preoperative clinical patient scanning. Phantom-less calibration could be employed; however, given that the Qualitative and Edge Detection techniques yielded the best agreement with the standard micro-CT measurements, improving estimations of BMD is expected to provide little value in cyst detection using clinical-CT. Nevertheless, our findings reinforce that clinical-CT can be used to reliably gauge the volume of cysts in glenohumeral OA.

Qualitative measurements are accurate and practical when the number and size of cysts within a subject is small; however, this is likely to be a labour-intensive technique with greater total cyst volumes. The semi-automated Edge Detection technique showed a relatively small bias and narrow limits of agreement in the Bland–Altman analysis, and a similar good to excellent reliability between graders as the Qualitative technique; overall providing comparable performance to Qualitative segmentation. Thus, semi-automated Edge Detection may be most useful in measuring cyst volume in glenohumeral OA, and likely in other joints as well.

Region Growing and Thresholding techniques may require less cyst-specific attention, but their accuracy is poor as indicated by high biases and wide limits of agreement in the Bland–Altman analysis. If not manually restricted to a region, Region Growing expands easily beyond cyst boundaries and can overestimate cyst volume. Thresholding measurements are limited by poorly defined and non-uniform cyst boundaries resulting from partial volume averaging. Thus, when lower thresholds are selected, voxels on the boundary of the cyst adjacent to surrounding bone may be excluded due to their higher intensity. Conversely, when higher thresholds are used, adjacent bone may be included in the segmentation and lead to overestimation of cyst volume. An automatic cyst segmentation technique using thresholding followed by morphology-based operations has been proposed for micro-CT [6, 7]; however, it is unlikely that this method could be accurately employed in clinical-CT due to these limitations of thresholding segmentation.

There were limitations associated with this study. First, the sample size was small, and no definite conclusions are possible based on these findings. However, this sample size is consistent with contemporary studies using micro-CT to look at bone [11], including those using Bland–Altman analysis [3]. An acceptable level of error for cyst volume measurements has not been clinically established, as the effects of subchondral bone cysts on clinical outcomes of glenohumeral OA are still being investigated. While Region Growing and Thresholding yielded less accurate and precise measurements, the lack of an acceptable level of error prevents interpreting whether discrepancies are clinically significant. Additionally, only one software program was used to evaluate the measurement techniques; however, other commercially available software programs tend to have comparable functionality based on common algorithms. Finally, the techniques evaluated in this study performed well in cystic OA with mild to moderate cystic presence; cases with large cystic presence, and/or where cyst boundaries are not clearly delineated were not evaluated.

## Conclusions

These findings provide preliminary evidence that Qualitative and Edge Detection segmentation techniques can be used on clinical-CT scans to accurately measure subchondral bone cyst volume in the glenohumeral joint, which may aid monitoring disease progression. Thus, there may be a potential use for these methods of quantifying cystic presence during treatment planning.

## Supplementary Information

**Additional file 1: Table S1.** Detailed descriptions of the measurement methods. Provides step-by-step descriptions of the measurement methods used in this study.

## Abbreviations

BMD: Bone Mineral Density
CI: Confidence Interval
CT: Computed Tomography
HU: Hounsfield Units
ICC: Intraclass Correlation Coefficient
OA: Osteoarthritis
RMSE: Root Mean Square Error
SD: Standard Deviation
