# Supplementary material for: Comparison of clinical-CT segmentation techniques for measuring subchondral bone cyst volume in glenohumeral osteoarthritis
Source: J Exp Orthop. 2023 Jan 6;10:1. doi: 10.1186/s40634-022-00564-x (PMC9823169; doi:10.1186/s40634-022-00564-x)
Supplement: Supplementary file 1 — Additional file 1: Table S1. Detailed descriptions of the measurement methods. Provides step-by-step descriptions of the measurement methods used in this study. [file 40634_2022_564_MOESM1_ESM.docx]

**Table S.1**. Detailed descriptions of the measurement methods

| **Segmentation Method** | **Description of the measurement method** |
| --- | --- |
| Qualitative | 1. Graders created the measurement by manually selecting voxels that they identified to be part of the cyst using the Edit mask draw tool. As cysts were defined as elliptical, spherical or irregularly shaped volumes of lower grayscale surrounded by an area of higher grayscale, graders based their qualitative measurements on the relative intensity of voxels compared to their surroundings. |
| Edge Detection | 1. A built-in 3D LiveWire tool was used to segment cysts based on manually placed points on multiple planes of the scan and automatically computed contours. 2. Graders manually placed points to indicate the border of the cyst on multiple slices of the coronal and axial planes. 3. Based on the placement of these points and automatically computed contours of the scan, the software then created a 3D segmentation of the cyst.    1. The automatically computed contours depended on 2 parameters: Gradient magnitude and Attraction coefficient. A high gradient magnitude value was used to attract the contour to brighter regions lying at the boundary of the cyst, as cysts are defined as lower grayscale surrounded by higher grayscale. A low attraction coefficient was used so that cavities near the boundaries of the object were taken into account, as cysts can be irregularly shaped. |
| Region Growing | 1. A cyst-specific threshold value was calculated by taking the mean midpoint HU value between cyst interior and adjacent bone. Graders calculated their own cyst-specific threshold value when taking measurements. 2. The calculated value was used as an upper threshold for segmentation. 3. Using the Region Grow tool, graders manually placed a seed point on a voxel within the threshold range on a middle slice of the cyst. All voxels within the threshold range that were connected on neighboring faces (6-connectivity) from this seed point were automatically isolated, creating the segmentation of the cyst. 4. In cases where the Region Growing tool could not fully isolate the cyst from surroundings, graders manually restricted the segmentation to include only slices containing the cyst. |
| Thresholding | 1. A threshold value of 392 HU, representing the mean cyst-specific midpoint intensity value from all Region Growing measurements, was used as a universal upper threshold value for measurements. 2. Using the Region Grow tool, graders manually placed a seed point on a voxel within the threshold range on a middle slice of the cyst. All voxels within the threshold range that were connected on neighboring faces (6-connectivity) from this seed point were automatically isolated, creating the segmentation of the cyst. 3. In cases where the Region Growing tool could not fully isolate the cyst from surroundings, graders manually restricted the segmentation to include only slices containing the cyst. |
